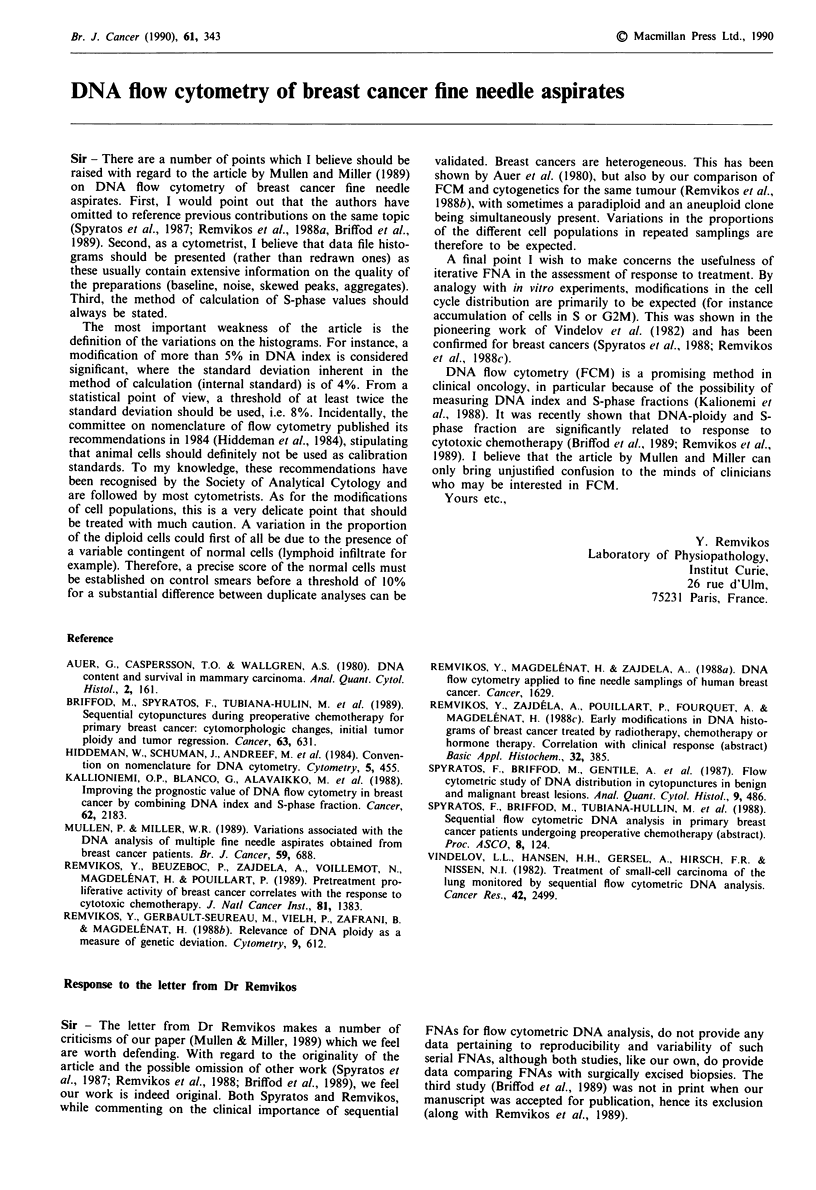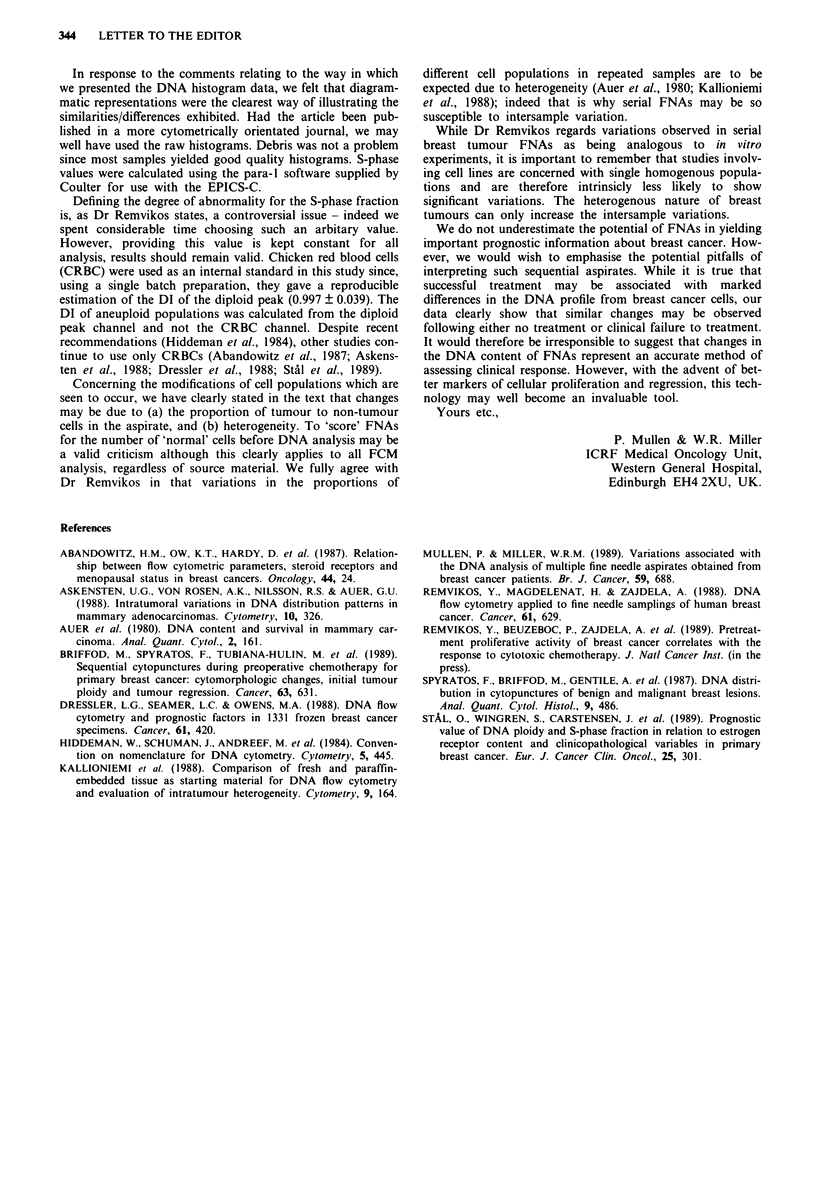# Response to the letter from Remvikos

**Published:** 1990-02

**Authors:** P. Mullen, W.R. Miller


**Response to the letter from Dr Remvikos**

**Sir** – The letter from Dr Remvikos makes a number of criticisms of our paper (Mullen & Miller, 1989) which we feel are worth defending. With regard to the originality of the article and the possible omission of other work (Spyratos *et al.*, 1987; Remvikos *et al.*, 1988; Briffod *et al.*, 1989), we feel our work is indeed original. Both Spyratos and Remvikos, while commenting on the clinical importance of sequential FNAs for flow cytometric DNA analysis, do not provide any data pertaining to reproducibility and variability of such serial FNAs, although both studies, like our own, do provide data comparing FNAs with surgically excised biopsies. The third study (Briffod *et al.*, 1989) was not in print when our manuscript was accepted for publication, hence its exclusion (along with Remvikos *et al.*, 1989).

In response to the comments relating to the way in which we presented the DNA histogram data, we felt that diagrammatic representations were the clearest way of illustrating the similarities/differences exhibited. Had the article been published in a more cytometrically orientated journal, we may well have used the raw histograms. Debris was not a problem since most samples yielded good quality histograms. S-phase values were calculated using the para-1 software supplied by Coulter for use with the EPICS-C.

Defining the degree of abnormality for the S-phase fraction is, as Dr Remvikos states, a controversial issue – indeed we spent considerable time choosing such an arbitary value. However, providing this value is kept constant for all analysis, results should remain valid. Chicken red blood cells (CRBC) were used as an internal standard in this study since, using a single batch preparation, they gave a reproducible estimation of the DI of the diploid peak ($0.997 \pm 0.039$). The DI of aneuploid populations was calculated from the diploid peak channel and not the CRBC channel. Despite recent recommendations (Hiddeman *et al.*, 1984), other studies continue to use only CRBCs (Abandowitz *et al.*, 1987; Askensten *et al.*, 1988; Dressler *et al.*, 1988; Stål *et al.*, 1989).

Concerning the modifications of cell populations which are seen to occur, we have clearly stated in the text that changes may be due to (a) the proportion of tumour to non-tumour cells in the aspirate, and (b) heterogeneity. To 'score' FNAs for the number of 'normal' cells before DNA analysis may be a valid criticism although this clearly applies to all FCM analysis, regardless of source material. We fully agree with Dr Remvikos in that variations in the proportions of different cell populations in repeated samples are to be expected due to heterogeneity (Auer *et al.*, 1980; Kallioniemi *et al.*, 1988); indeed that is why serial FNAs may be so susceptible to intersample variation.

While Dr Remvikos regards variations observed in serial breast tumour FNAs as being analogous to *in vitro* experiments, it is important to remember that studies involving cell lines are concerned with single homogenous populations and are therefore intrinsicly less likely to show significant variations. The heterogenous nature of breast tumours can only increase the intersample variations.

We do not underestimate the potential of FNAs in yielding important prognostic information about breast cancer. However, we would wish to emphasise the potential pitfalls of interpreting such sequential aspirates. While it is true that successful treatment may be associated with marked differences in the DNA profile from breast cancer cells, our data clearly show that similar changes may be observed following either no treatment or clinical failure to treatment. It would therefore be irresponsible to suggest that changes in the DNA content of FNAs represent an accurate method of assessing clinical response. However, with the advent of better markers of cellular proliferation and regression, this technology may well become an invaluable tool.

Yours etc.,

P. Mullen & W.R. Miller
ICRF Medical Oncology Unit,
Western General Hospital,
Edinburgh EH4 2XU, UK.

### References

ABANDOWITZ, H.M., OW, K.T., HARDY, D. *et al.* (1987). Relationship between flow cytometric parameters, steroid receptors and menopausal status in breast cancers. *Oncology*, **44**, 24.

ASKENSTEN, U.G., VON ROSEN, A.K., NILSSON, R.S. & AUER, G.U. (1988). Intratumoral variations in DNA distribution patterns in mammary adenocarcinomas. *Cytometry*, **10**, 326.

AUER *et al.* (1980). DNA content and survival in mammary carcinoma. *Anal. Quant. Cytol.*, **2**, 161.

BRIFFOD, M., SPYRATOS, F., TUBIANA-HULIN, M. *et al.* (1989). Sequential cytopunctures during preoperative chemotherapy for primary breast cancer: cytomorphologic changes, initial tumour ploidy and tumour regression. *Cancer*, **63**, 631.

DRESSLER, L.G., SEAMER, L.C. & OWENS, M.A. (1988). DNA flow cytometry and prognostic factors in 1331 frozen breast cancer specimens. *Cancer*, **61**, 420.

HIDDEMAN, W., SCHUMAN, J., ANDREEF, M. *et al.* (1984). Convention on nomenclature for DNA cytometry. *Cytometry*, **5**, 445.

KALLIONIEMI *et al.* (1988). Comparison of fresh and paraffin-embedded tissue as starting material for DNA flow cytometry and evaluation of intratumour heterogeneity. *Cytometry*, **9**, 164.

MULLEN, P. & MILLER, W.R.M. (1989). Variations associated with the DNA analysis of multiple fine needle aspirates obtained from breast cancer patients. *Br. J. Cancer*, **59**, 688.

REMVIKOS, Y., MAGDELENAT, H. & ZAJDELA, A. (1988). DNA flow cytometry applied to fine needle samplings of human breast cancer. *Cancer*, **61**, 629.

REMVIKOS, Y., BEUZEBOC, P., ZAJDELA, A. *et al.* (1989). Pretreatment proliferative activity of breast cancer correlates with the response to cytotoxic chemotherapy. *J. Natl Cancer Inst.* (in the press).

SPYRATOS, F., BRIFFOD, M., GENTILE, A. *et al.* (1987). DNA distribution in cytopunctures of benign and malignant breast lesions. *Anal. Quant. Cytol. Histol.*, **9**, 486.

STÅL, O., WINGREN, S., CARSTENSEN, J. *et al.* (1989). Prognostic value of DNA ploidy and S-phase fraction in relation to estrogen receptor content and clinicopathological variables in primary breast cancer. *Eur. J. Cancer Clin. Oncol.*, **25**, 301.